# AUGS-PERFORM: A New Patient-Reported Outcome Measure to Assess Quality of Prolapse Care

**DOI:** 10.1097/SPV.0000000000001225

**Published:** 2022-06-22

**Authors:** Michele O’Shea, Sarah Boyles, Catherine S. Bradley, Kristin Jacobs, Molly McFatrich, Vivian Sung, Kevin Weinfurt, Nazema Y. Siddiqui

**Affiliations:** From the ∗ Duke University Health System, Durham, NC; † The Oregon Clinic, Portland, OR; ‡ University of Iowa Carver College of Medicine, Iowa City, IA; § Rush University, Chicago, IL; ∥ Duke University Medical Center, Durham, NC; ¶ Alpert Medical School of Brown University, Providence, RI.

**Keywords:** quality, pelvic organ prolapse, patient-reported outcomes

## Abstract

Patient-reported outcomes (PRO) are important for measuring quality of care, particularly for interventions aimed at improving symptom bother such as procedures for pelvic organ prolapse. We aimed to create a concise yet comprehensive PRO measurement tool to assess pelvic organ prolapse care in high-volume clinical environments.

**Methods:**

The relevant concepts to measure prolapse treatment quality were first established through literature review, qualitative interviews, and a patient and provider-driven consensus-building process. Extant items mapping to these concepts, or domains, were identified from an existing pool of patient-reported symptoms and condition-specific and generic health-related quality of life measures. Item classification was performed to group items assessing similar concepts while eliminating items that were redundant, inconsistent with domains, or overly complex. A consensus meeting was held in March 2020 where patient and provider working groups ranked the remaining candidate items in order of relevance to measure prolapse treatment quality. After subsequent expert review, the revised candidate items underwent cognitive interview testing and were further refined.

**Results:**

Fifteen relevant PRO instruments were initially identified, and 358 items were considered for inclusion. After 2 iterative consensus reviews and 4 rounds of cognitive interviewing with 19 patients, 11 final candidate items were identified. These items map 5 consensus-based domains that include awareness and bother from prolapse, physical function, physical discomfort during sexual activity, pain, and urinary/defecatory symptoms.

**Conclusions:**

We present a concise set of candidate items that were developed using rigorous patient-centered methodology and a national consensus process, including urogynecologic patients and providers.

Pelvic organ prolapse can be appreciated in up to 50% of women undergoing pelvic examination, although only a minority are symptomatic and require intervention.^1^ Nonetheless, an individual woman has a 1 in 8 risk of undergoing surgery for prolapse by age 80 years.^2^ While a multitude of comparative effectiveness trials have been performed that tested many different surgical treatments for prolapse, there has been considerable heterogeneity in the metrics used to define “success.”

Currently available quality indicators for tracking and improving surgical care for women with prolapse are primarily limited to process measures, such as whether antibiotics were given,^3^ or rare complications, such as whether organ injury occurred.^4^ Although these indicators are important components of measuring overall surgical quality, deviations happen rarely for high-volume low-risk procedures such as those used for prolapse. Furthermore, process measures do not comprehensively address all facets of high-quality care for prolapse. Importantly, the principal reason to perform surgery for prolapse is to improve a woman’s subjective bother associated with the prolapse, rather than any objective measurement of the prolapse itself. Thus, measuring and tracking patient-reported outcomes (PRO), including prolapse-related symptoms, bother, and functional impact, is critical to fully evaluate the surgical care provided. Assessing these important outcomes is presently challenging because we lack rigorous PRO tools that encompass multiple important domains, or concepts that capture patient-important outcomes, in a concise yet comprehensive format.

In 2016, the American Urogynecologic Society (AUGS) convened a consensus conference focused on identifying critical areas of need for prolapse research.^5^ One of the conference conclusions was the critical need for PRO measures for prolapse and a standardized group of prolapse outcomes that could be used routinely in large trials, community-based research, and quality metric development. A follow-up conference in 2019 confirmed this need and created a roadmap for creation of a standardized PRO-based quality measure for prolapse treatment.^6^ Our present objective is to describe how this roadmap was used to develop a concise yet comprehensive PRO quality assessment tool for surgical and nonsurgical treatment of prolapse, named AUGS PERFORM (AUGS PERFORmance Measure).

## MATERIALS AND METHODS

### Identification of Domains

**FIGURE 1:**
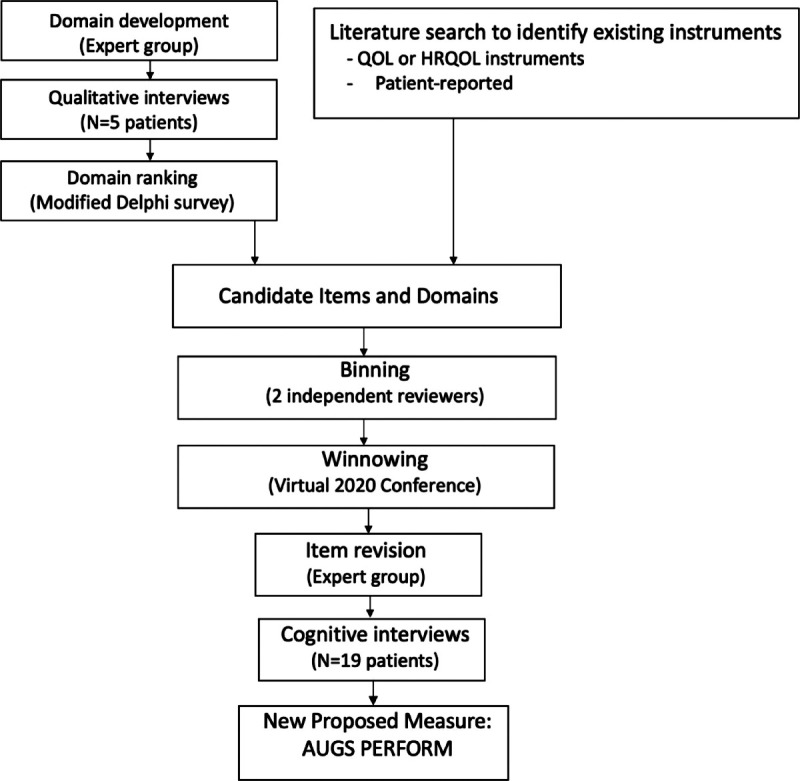
Overview of item development process. QOL, quality of life; HRQOL, health-related quality of life.

**FIGURE 2:**
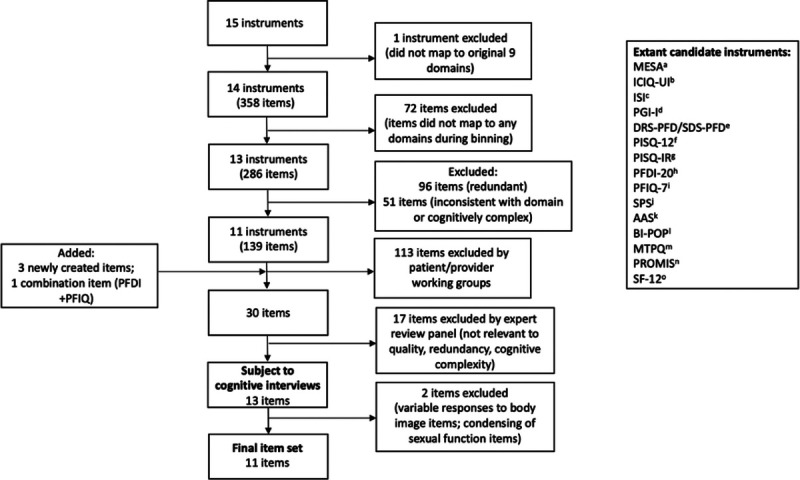
Candidate instrument and item selection. A, Medical Epidemiologic and Social Aspects of Aging Incontinence Questionnaire.^10^ B, International Consultation on Incontinence-Urinary Incontinence, Short Form.^11^ C, Sandvik-Hunskaar Incontinence Severity Index.^12^ D, Patient Global Impression of Improvement.^13,14^ E, Decision-Regret Scale-Pelvic Floor Disorders and Satisfaction with Decision Scale-Pelvic Floor Disorders.^15–17^ F, Pelvic Organ Prolapse/Urinary Incontinence Sexual Questionnaire-12.^18^ G, Pelvic Organ Prolapse/Urinary Incontinence Sexual Questionnaire—IUGA Revised.^19^ H, Pelvic Floor Distress Inventory-20.^20^ I, Pelvic Floor Impact Questionnaire-7.^20^ J, Surgical Pain Scales.^21^ K, Activities Assessment Scale.^22^ L, Body Image and Pelvic Organ Prolapse Questionnaire.^23^ M, Modified TOMUS Pain Questionnaire. N, Patient-Reported Outcomes Measurement Information System.^24^ O, Short Form-12 Health Survey.^25^ PFDI, Pelvic Floor Distress Inventory; PFIQ, Pelvic Floor Impact Questionnaire.

**FIGURE 3:**
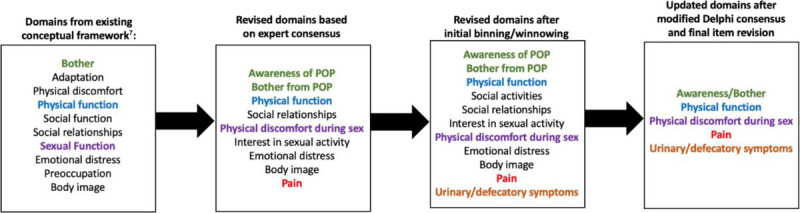
Domain development process. Sung VW, Rogers RG, Barber MD, Clark MA. Conceptual framework for patient-important treatment outcomes for pelvic organ prolapse. *Neurourol Urodyn*. 2014;33(4):414–419. doi:10.1002/nau.22397.

A general overview of the item development process is presented in Figure 1. Before determining the relevant PRO items to include, it was first necessary to define a set of domains capturing the relevant concepts to be measured to assess prolapse treatment quality. To determine a set of initial candidate domains representing the patient-important aspects of the prolapse experience and prolapse treatment, we referred to a previously published conceptual framework for prolapse derived from patient focus groups.^7–9^ An expert group consisting of 3 urogynecologists (VS, NS, MO) and one health measurement expert (KW) reviewed the initial set of 10 candidate domains derived from the previously published conceptual framework for prolapse (Fig. 2 and Fig. 3).^7^ The domains comprised the overarching areas of physical health, mental health, social health, and vaginal bulge symptoms.^7^ These domains were then further refined by the expert group to maximize conceptual clarity after review of the original focus group discussion transcripts. Standard definitions were also agreed upon for each domain.

The original domains under consideration were drawn from a population of predominantly White, college-educated women,^7^ and thus perspectives from more diverse racial and educational backgrounds were sought through additional qualitative interviews with 5 participants recruited from Duke University Medical Center in Durham, North Carolina. The interviews were performed by a trained interviewer experienced in qualitative data collection for the purposes of PRO instrument development. All interview participants were non-Hispanic Black, and mean age of participants was 72.8 ± 12.0 years. A semistructured interview guide was used to confirm whether the original candidate domains were also deemed important by a more diverse population and to elicit any additional concepts that were not previously identified. A total of 5 additional interviews were estimated to be required to achieve concept saturation.

After completion of qualitative interviews, the updated candidate domains (Fig. 3) were then prioritized using a modified Delphi method to create consensus upon the importance of each domain with respect to the measurement of prolapse treatment quality. A broad group of urogynecologic providers and patient representatives participated in this modified Delphi process, which generated a refined set of domains that were considered during patient and provider working groups at a consensus conference held in March 2020.^26^

### Identification of Extant Items

Rather than create a set of de novo PRO questionnaire items, an existing pool of validated questionnaires, including condition-specific and generic health-related quality of life measures, was identified via a literature search conducted and reviewed by 5 expert clinicians who were members of the AUGS Quality Committee. Each item was reviewed for intended patient population, literacy level, parent project and reason created, previous validation, and level of patient input during instrument development.

### Binning

To determine which of the extant questionnaire items from each instrument identified previously “mapped” to each of the updated candidate domains, we proceeded with the process of binning, which refers to the systematic grouping of candidate items (often from different parent instruments) based on the concept each item appears to measure.^27^ Bins were not determined a priori nor were a target number of items to be selected for each bin. Rather, all bins were derived organically from the systematic grouping of extant items.

Two members of the working group (M.O. and K.J.) compiled and independently binned all candidate questionnaire items. Discrepancies in bins or item categorization were discussed and resolved by the 2 reviewers. The grouping of similar items within bins allowed identification of redundant items, which were then removed. When 2 or more items were considered redundant, the item with the greatest cognitive clarity based on consensus was retained, regardless of the source instrument for that item. Items that were not wholly appropriate for a bin but were still considered to measure concepts relevant to prolapse treatment were marked for discussion with the remainder of the working group.

### Winnowing

After binning, 139 candidate items remained (Fig. 2). The goal of winnowing was to further condense these to a final set of items, which could be tested in cognitive interviews. Working groups of providers and patient representatives reviewed the candidate items at the virtually held AUGS Prolapse Consensus Conference in March 2020. All AUGS members were invited to attend a virtual meeting via emailed invitation, whereas patient representatives were invited through the AUGS Patient Advisory Group. Demographic data of conference participants were not collected. During the conference, virtual breakout sessions were created for working groups of provider and patient representatives for each domain or cluster of domains. Working groups were moderated by 2 experts from either urogynecology or health measurement fields. For each domain, participants reviewed and discussed each candidate item binned within the domain to achieve consensus in ranking candidate items in order of importance, from “obvious winners” and “top-tier items” to “middle-tier” and “lowest-tier” items. Adapted from the Patient-Reported Outcomes Measurement Information System (PROMIS) methodology,^27^ the following criteria were used for reviewing items: (1) consistency with the domain definition, (2) semantic redundancy with another item, (3) universal applicability of the item, (4) applicability of the stem to prolapse, and (5) cognitive complexity of the item.^27^ Items were also revised as needed to improve clarity, simplicity, and consistency with the domain definition. Novel items were also generated by the working groups when a relevant construct was felt not to be adequately captured by any of the extant items.

### Item Revision

At the completion of the binning and winnowing process, a panel of 5 experts in urogynecology and 1 expert in health measurement reviewed the list of candidate items to assess face and content validity, comprehensiveness, literacy level, item wording, and response options. Language deemed cognitively complex was amended to optimize patient comprehension before item testing. Items belonging to domains that were deemed to be “minimally relevant” for the purposes of measuring prolapse treatment quality were identified through a modified Delphi method and were eliminated. Possible response options were also reviewed by the panel and modified to minimize respondent burden.

Each item and its corresponding domain were once again reviewed by the expert panel specifically for their utility in assessing prolapse treatment quality. Items that were deemed important concepts in the experience of living with prolapse, but not particularly useful for measuring treatment quality, were eliminated.

### Cognitive Interviews

Although some of the items for testing had been previously tested through the rigorous PROMIS item development process,^27^ the content validity of most candidate items had not been assessed via cognitive testing. Therefore, we conducted qualitative interviews with patients using a semistructured interview guide. Interview probes included questions such as, “What do you think this question is asking in your own words?” or “How did you decide <response> was the best option for you?” Through this process, we obtained patient feedback on item wording, recall period, and response options.^28^ Given that cognitive interviews were conducted during the setting of the severe acute respiratory syndrome coronavirus 2 pandemic, participants were asked as to whether their responses were altered by social distancing measures or physical activity restrictions enacted to prevent severe acute respiratory syndrome coronavirus 2 transmission. All interviews were conducted via telephone and audio recorded with participant permission.

Trained interviewers used a retrospective probing technique during cognitive interviews. In advance of their interview, participants were mailed a paper copy of the draft questionnaire. While on the phone, participants were asked to complete the questionnaire and then to explain their responses to particular items. While completing the questionnaire, participants were also asked to mark items they thought were hard to understand.

Four rounds of cognitive interviews were conducted. In the first round, participants reviewed all questions in the measure. In rounds 2–4 of interviews, participants reviewed only those questions that were significantly revised from round 1. Items were defined as being “significantly revised” if their revision involved (1) adding or removing a word(s) that changes the meaning of a phrase, (2) word substitutions that in the judgment of the investigators are more than a semantic simplification, or (3) significant changes to the response options (eg, changing from a severity to a frequency scale). The sample size of 5 participants per round is consistent with cognitive interview guidelines.^28^

Participants were recruited from a pool of participants who had previously participated in a Pelvic Floor Disorders Network trial through Duke University and had given permission to be contacted in the future for further research study participation. Additional participants were recruited from the Duke University Urogynecology Clinic. Treating urogynecologic surgeons and nurse practitioners initially approached patients about participation in the study, and a member of study personnel later contacted interested patients to provide detailed information about interview participation. Efforts were made to recruit patients from diverse educational and racial/ethnic backgrounds, as well as patients who were presurgery and postsurgery, to ensure that responses to cognitive interviews were from a more broadly representative patient population. Upon interview completion, participants received $50 remuneration.

### Final Item Revision

Each round of the cognitive interviewing process resulted in a refined set of candidate items based on patient input, which were then reviewed by the expert panel. Additional revisions were made to further optimize item clarity and order of items, as well as remove items that belonged to a domain deemed to be less critically important by the modified Delphi process. After the final round of cognitive interviews, response options were reviewed a final time for overall consistency. Public comment was solicited via an online link to AUGS members, which was open for a 30-day window in November 2021.

## RESULTS

### Domain Determination

Figure 3 provides the evolution of candidate domains from the original domains identified by the Sung et al^27^ conceptual framework. After broad input by providers and patient representatives, 5 final domains were deemed “critically important” to the assessment of quality of prolapse treatment. A total of 56 responses representing 2 rounds of the modified Delphi process were represented.

### Identification of Extant Items

Fourteen existing instruments were included, which were able to be mapped to the 10 original domains described by Sung et al.^7^ Notably, although some instruments were developed using a satisfactory patient-centered methodology, most PRO instruments were not developed in this manner (Fig. 2).

### Binning and Winnowing

A total of 286 of the original 358 candidate PRO items were mapped to the 9 revised expert consensus domains (Fig. 2). Ninety-six items were removed for redundancy; 51 were removed for inconsistency with a predetermined domain or excessive cognitive complexity, leaving 139 candidate items. During the binning process, 2 new domains (pelvic pain and urinary/defecatory symptoms) were identified that had not been previously described by the existing conceptual framework but were deemed to represent potentially important concepts for clinically relevant and patient-important treatment outcomes.

The winnowing process also resulted in further elimination of individual items or entire bins that, although important components of the experience of living with prolapse were not deemed to be useful concepts to specifically assess prolapse treatment quality. For example, although emotional distress is an extremely important concept related to the prolapse experience, the concept of emotional distress is not necessarily a useful measure with which to assess the quality of treatment provided. This is because of the distinction between meeting an outcome measure versus meeting a performance measure—in which the latter can only be assessed in terms of outcomes that are reasonably related to the quality of a surgeon’s care (such as the absence of bulge symptoms or de novo dyspareunia).

After the virtual breakout sessions from the March 2020 prolapse consensus conference, there were 30 items representing 10 domains that were categorized by working groups into “obvious winners” and “top-tier items” categories (Fig. 2). These included 3 new items created during the sessions and 6 items, which were modifications of existing items.

### Item Revision

The final 30 items prioritized by conference working groups were drawn from a heterogeneous set of instruments of varying methodologies, including several novel items generated at the conference. Thus, they had considerable variations in wording style, recall time frame, response options, and literacy level. Response options were modified where needed to maximize consistency with each item stem and with the remainder of the questionnaire items. While the optimal period of interest is currently unknown for assessing PRO measures after prolapse treatment, it was determined that a 30-day recall period for most items would be reasonable for 2 reasons. First, given that some symptoms associated with prolapse (such as constipation) may fluctuate on a daily or weekly basis, the patients’ overall experience over a 30-day period would provide a more informative picture of health status than a shorter (eg, 7-day) period. Second, prior studies have demonstrated surprisingly high correlations between 7- and 30-day recall periods for items measuring sexual function^29^ and lower urinary tract symptoms.^30^

Each item and its corresponding domain were once again reviewed by the expert panel specifically for their utility in assessing prolapse treatment quality. Items that were deemed important concepts in the experience of living with prolapse, but not particularly useful for measuring treatment quality, were eliminated. Seventeen items were ultimately eliminated by the expert panel because of redundancy with other items, cognitive complexity, or irrelevance to the measurement of prolapse treatment quality, leaving 13 items for cognitive interview testing.

### Cognitive Interviews

A total of 13 items mapping to 6 domains were tested during cognitive interviews (Fig. 2). Nineteen participants participated in 4 rounds of cognitive interviews, with new participants participating in each iteration of the cognitive interviews. The mean age of cognitive interview participants was 65.7 ± 12.4 years. Seven participants (37%) identified as non-Hispanic Black and 12 (63%) as non-Hispanic White. Three of the cognitive interview participants (16%) were sexually active. Eleven participants (57%) had previously undergone prolapse surgery, whereas 6 participants (32%) were anticipating surgery. Two participants (11%) were pessary users. All stages of prolapse were represented among the cognitive interview participants, with 3 participants having stage 0, 3 participants with stage I, 6 participants with stage II, 6 participants with stage III, and 1 participant with stage IV prolapse.

Overall, the concept of prolapse awareness and bother was well understood by participants. Items on urinary and defecatory symptoms^31^ as well as items on physical function^32,33^ and pelvic pain^34^ derived from the Pelvic Floor Distress Inventory and PROMIS were well understood. Items related to body image were initially tested but eventually excluded because of highly variable responses during cognitive interviews and inconsistent correlation with prolapse or improvement after prolapse surgery in other PRO studies. For example, although patients with advanced prolapse were previously reported to have poorer body image compared with controls, overall body image was not significantly different.^35^ Another study found that only 40% of patients had achieved the goal of improving body image at 10 years postoperatively, compared with 90% who had achieved the goal of symptom relief.^36^ Despite being an important component of the prolapse experience, given the complex multifactorial nature of body image, it was determined that the concept would be challenging to incorporate as a universal performance measure. Sexual function items also required multiple rounds of revisions and retesting primarily due to difficulties in distinguishing the concept of limiting sexual activity due to prolapse or genital pain from sexual inactivity due to other reasons (such as lack of a partner). At the completion of cognitive interviews, a set of 11 items mapping to 5 remaining domains was proposed for the final questionnaire (Table 1; Supplementary Table 1, http://links.lww.com/FPMRS/A336). Nineteen AUGS members provided public comment during the 30-day window. After review and consideration of individual item feedback, substantive changes to the instrument were ultimately not required.

**TABLE 1:**
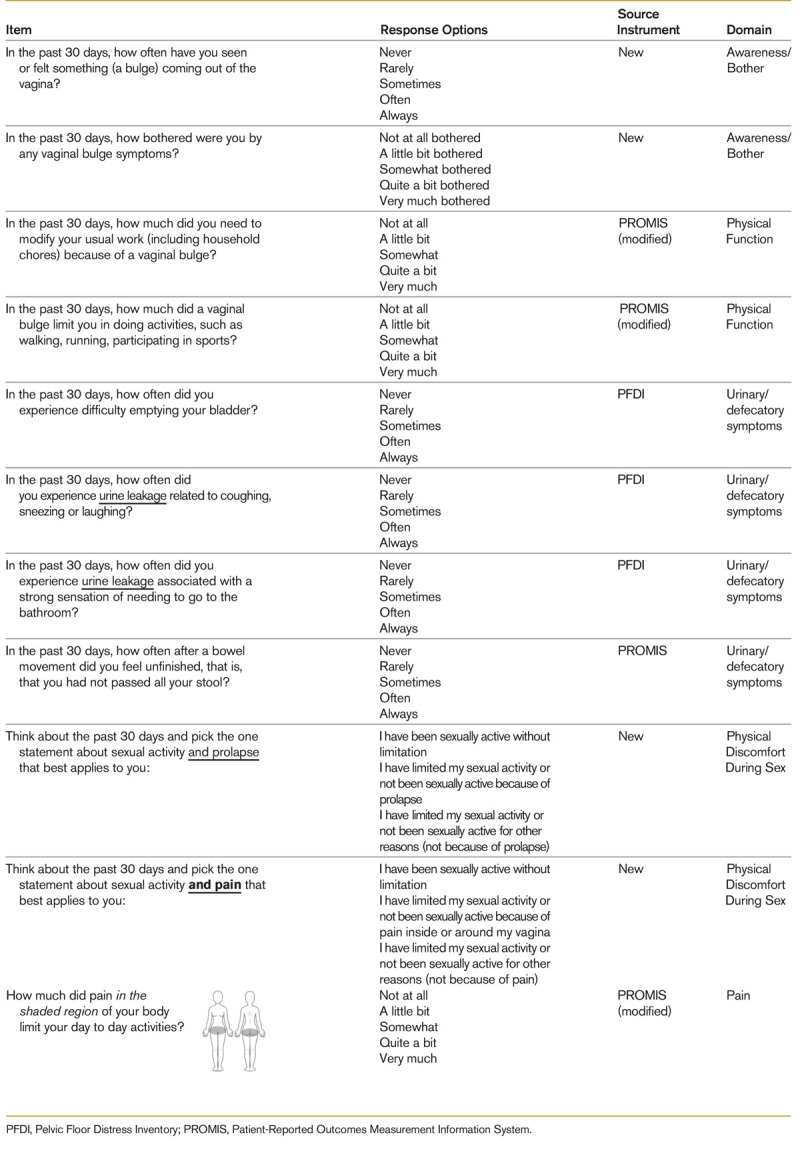
Final Items With Source Instruments and Domains

## DISCUSSION

With this proposed set of PRO items, we aim to fill an existing gap in the field of pelvic reconstructive surgery with the creation of an instrument to aid in the efficient measurement of prolapse treatment quality. The strength of the present PRO lies in its development using rigorous, patient-centered methodology consistent with the NIH PROMIS approach.^31^ The items are consistent with concepts elicited by experts, providers, and patient representatives and were agreed upon as being most critical to assess the quality of prolapse treatment by a national consensus-building process. Furthermore, the items were also generally well received by members of the AUGS community.

A challenge for assessing prolapse with PRO measures is that multiple, sometimes lengthy, instruments would be required to measure the full range of patient-centric domains. Several domains from the original conceptual framework were eliminated at the conclusion of the consensus-building and item revision processes. Although some domains were not considered to be as critically relevant during the modified Delphi process (such as social relationships and social activities), others were ultimately eliminated because of being deemed inappropriate measures of treatment quality and beyond a surgeon or treating provider’s control. For example, emotional distress and sexual interest, while clearly important concepts to patients living with prolapse,^7,8,37^ tend to have chronic multifactorial etiologies, many of which may be not be solely influenced by prolapse treatment. Similarly, we recognize that postoperative pain may be affected by factors outside of a treating provider’s control. However, given that de novo chronic pain and de novo dyspareunia have been reported to result from prolapse surgery, pain was ultimately thought to be important to include as an outcome measure.^38^

What patients perceive as severe complications do not always correspond with surgeons’ traditional perceptions of severe complications and, therefore, the patient perspective is essential when designing measures to track the quality of care.^39^ For example, although urinary and defecatory symptoms do not always correlate with anatomic prolapse findings, patients tend to perceive de novo or worsening constipation and urinary incontinence as being severe adverse events.^40–44^ Not surprisingly, these symptoms were found to be critically relevant during our consensus process. Furthermore, given that urinary and defecatory symptoms could both contribute to the decision to undergo surgery and be affected by the prolapse surgery itself, the domain was regarded as being a highly critical concept to measure and track.

While many candidate items from prolapse-specific PRO instruments were included for consideration, items originating from PROMIS instruments represented the majority of items surviving the binning and winnowing process. This is likely a testament to the strength of the patient-centered methodology that led to these items being conceptually clear and patient-friendly with respect to cognitive complexity, allowing them to outperform many prolapse-specific instrument items measuring similar concepts.

Strengths of our process include capitalizing on extant condition-specific PRO items and previously validated general health-related quality of life measures that had previously been developed using rigorous patient-centered methodology. Another strength was the inclusion of patients in every step of the development process, including concept elicitation, consensus-building on domains, the winnowing process for item development, and cognitive interviewing of candidate items.

Our work had several limitations. First, the testing of the candidate items via cognitive interviews was conducted at a single institution in the southeastern region of the United States, which limits the overall diversity of patients and perspectives represented in the interviews. While we aimed to recruit a diverse cohort of patients during the 19 cognitive interviews, the representation of exclusively non-Hispanic Black and non-Hispanic White participants reflects the predominant racial and ethnic makeup of patients at the recruitment site. Further psychometric evaluation of this instrument is necessary in larger populations of patients with diverse demographic, racial, and ethnic characteristics from different geographic regions. Another limitation is that nonsexually active and older patients were oversampled in our population of cognitive interview patients. While items performed well among the 3 patients who were sexually active, further validation of these items among a larger population of younger and sexually active patients is needed.

In conclusion, we present a concise candidate PRO for quality assessment that was developed using rigorous patient-centered methodology and using a consensus process between urogynecologic patients and providers. Because the paradigm for assessing health care quality was first described to include structural, process, and outcome measures, the universal assessment of patient-reported functional health outcomes has yet to be realized in our field.^45^ We are optimistic that the current set of items will ultimately aid in the efficient tracking of quality of prolapse care while focusing on the concepts most relevant to our patients. Efforts are underway to evaluate these candidate items via field testing for psychometric properties. Before broad-scale use, AUGS-PERFORM requires rigorous validation and assessment of responsiveness to change. Should this instrument demonstrate these characteristics, it may ultimately be useful for incorporation into large-scale urogynecologic quality improvement programs.

## Supplementary Material
