# Supplementary material for: AUGS-PERFORM: A New Patient-Reported Outcome Measure to Assess Quality of Prolapse Care
Source: Female Pelvic Med Reconstr Surg. 2022 Jun 22;28(8):468–78. doi: 10.1097/SPV.0000000000001225 (PMC9365262; doi:10.1097/SPV.0000000000001225)
Supplement: Supplementary file 1 [file jpms-28-468-s001.docx]

**APPENDIX**

**Supplementary Table 1:** Patient-reported outcome items revised from Round 1 to Round 4

| **Domain** | **Round 1**  **Wording** | **Round 2 Wording** | **Round 3 Wording** | **Round 4 Wording** | **Reason for Change** |
| --- | --- | --- | --- | --- | --- |
| **POP Awareness and bother** | In the past 30 days, how often have you seen or felt something (a bulge) coming out of the vagina? | [no change from Round 1] | [no change from Round 2] | [no change from Round 3] | N/A – original wording well understood |
| **POP Awareness and bother** | In the past 30 days, how bothered were you by any vaginal bulge symptoms? | [no change from Round 1] | [no change from Round 2] | [no change from Round 3] | N/A – original wording well understood  Final meeting with expert panel after **Round 4**: 🡪 Response options modified to harmonize with remainder of items |
| **Body image** | How comfortable are you with the appearance and feeling of your vagina? | How comfortable are you with the appearance of your genital area? | How happy are you with the appearance of your genital area? | *[Removed]* | **Round 1**: Comfort inconsistently interpreted as physical versus emotional. ‘Appearance’ interpreted as visual appearance, feeling = physical feeling (that can be touched or felt), therefore the word ‘feeling’ dropped for clarity. ‘Genital area’ also better understood than vagina which is thought to be more internal structure, and thus changed to ‘genital area.’  **Round 2**: Issues with interpretation of ‘comfortable’ as physical or emotional. Therefore group agreed to attempt simplifying with emotional response of ‘happy’  **Round 3**: Extremes of responses in participants who never really thought about /looked at genital appearance vs those who immediately identified themselves as bothered  **Meeting 1/13:** Delete due to disparate responses based on age/culture; not useful for broad use in patient population to assess quality of care |
| **Physical Discomfort During Sexual Activity (prolapse)** | In the past 30 days, did you have any type of sexual activity? (Examples of sexual activity are masturbation, oral sex, and sexual intercourse.)   Yes   No  There are many reasons why people may not have had sexual activity during the month. What are the reasons why you did not have sexual activity in the past 30 days? Please read the list carefully and check every reason that applies to you, even if it happened only one time during the past 30 days   Was not interested in having sexual activity   Dryness or pain in or around my vagina   Difficulties with orgasm/climax   Don't enjoy sexual activity   Due to bladder or bowel problems   Due to a prolapse (bulge in the vaginal area)   Other health condition   No partner, or partner not available/unable to have sex   Some other reason (Specify) | In the past 30 days, did you have any type of sexual activity? (Examples of sexual activity are masturbation, oral sex, and sexual intercourse.)   Yes   No  There are many reasons why people may not have had sexual activity **or** limited their sexual activity during the last month. If this applies to you, what are the reasons why you did not have sexual activity **or** limited your sexual activity in the past 30 days? Please read the list carefully and check every reason that applies to you, even if it happened only one time during the past 30 days   Not applicable/Have not limited sexual activity in the past 30 days   Was not interested in having sexual activity   Dryness or pain in or around my vagina   Difficulties with orgasm/climax   Don't enjoy sexual activity   Due to bladder or bowel problems   Due to a prolapse (bulge in the vaginal area)   Other health condition   No partner, or partner not available/unable to have sex   Some other reason (Specify): | In the past 30 days, have you limited your sexual activity? (Examples of sexual activity are masturbation, oral sex, and sexual intercourse.)   No - I have not limited sexual activity (skip to question 7)   No – I am not sexually active (skip to question 7)   Yes – I have limited sexual activity  There are many reasons why people may not have had sexual activity **or** limited their sexual activity during the last month. If this applies to you, what are the reasons why you did not have sexual activity **or** limited your sexual activity in the past 30 days? Please read the list carefully and check every reason that applies to you, even if it happened only one time during the past 30 days   Dryness or pain in or around my vagina   Due to bladder or bowel problems   Due to a prolapse (bulge in the vaginal area)   Some other reason (Specify): | A) In the past 30 days, have you limited your sexual activity or not been sexually active because of prolapse? Please select the one option that best applies:   I have limited sexual activity or not been sexually active because of prolapse   I have limited sexual activity or not been sexually active for another reason (not because of prolapse)   I have not limited my sexual activity  B) In the past 30 days, have you changed your sexual activity or not been sexually active because of prolapse? Please select the one option that best applies:   I have changed my sexual activity or not been sexually active because of prolapse   I have changed my sexual activity or not been sexually active for another reason (not because of prolapse)   I have not changed my sexual activity  C) Think about the past 30 days and pick the one statement about sexual activity **and prolapse** that best applies to you:   I have been sexually active without limitation   I have limited my sexual activity or not been sexually active because of prolapse   I have limited my sexual activity or not been sexually active for other reasons (not because of prolapse) | **Round 1:** Answer options simplified to condense those related to partner; also allow option to say NA if not limited sexual activity in past 30 days  **Round 2:** Group decided to create a skip pattern as pts who are not sexually active or did NOT limit sexual activity would not apply to following question on ‘why’ sexual activity was limited. Response options were simplified to just those related to POP and pain – from surgical quality standpoint would not be necessarily relevant the fact that their partner has medical issues/marital problems, etc  **Round 3:** Clarify question on whether patients have limited sexual activity because of prolapse (because that is ultimately what surgeon can reasonably be responsible for), and eliminate other reasons that are less likely to be corrected by surgeon (dryness, bladder/bowel issues, etc). However, still needed a leading statement on the types of ‘sexual activity’ referred to, as many participants interpreted ‘sexual activity’ to only include intercourse.  **Round 4**: Patients answered consistently across both POP and pain-related questions  1 participant who was not sexually active because of POP answered only on C that she had limited because of prolapse. She interpreted ‘limited’ as not being sexually active at all but answered appropriately in version C. Ultimately it was decided that additional interviews were unlikely to change the preference for Version C given that patients were all able to respond in accordance with sexual activity status to this version. |
| **Physical Discomfort During Sexual Activity (pain)** | In the past 30 days, how often have you had pain inside or around your vagina with sexual activity?   Never   Rarely   Sometimes   Often   Always | In the past 30 days, how often have you had pain inside or around your vagina with sexual activity?   Not applicable/Have not had sexual activity in the past 30 days   Never   Rarely   Sometimes   Often   Always | In the past 30 days, how often have you had pain inside or around your vagina with sexual activity?   Not applicable/Have not had sexual activity in the past 30 days   Never   Rarely   Sometimes   Often   Always | A) In the past 30 days, have you limited your sexual activity or not been sexually active because of pain inside or around your vagina? Please select the one option that best applies:   I have limited sexual activity or not been sexually active because of pain inside or around my vagina   I have limited sexual activity or not been sexually active for another reason (not because of pain)   I have not limited my sexual activity  B) In the past 30 days, have you changed your sexual activity or not been sexually active because of pain inside or around your vagina? Please select the one option that best applies:   I have changed my sexual activity or not been sexually active because of pain inside or around my vagina   I have changed my sexual activity or not been sexually active for another reason (not because of pain)   I have not changed my sexual activity  C) Think about the past 30 days and pick the one statement about sexual activity **and pain** that best applies to you:   I have been sexually active without limitation   I have limited my sexual activity or not been sexually active because of pain inside or around my vagina   I have limited my sexual activity or not been sexually active for other reasons (not because of pain) | **Round 1**: Overall difficult to determine whether participants answer ‘never’ because they don’t have pain or because haven’t been sexually active. Thus a ‘NA—have not had sexual activity’ answer option added.  **Round 2**: Moved pain question to being the first question on sex, prior to asking about ‘limiting’ sexual activity. Rationale: patients may have pain yet NOT LIMIT sexual activity  **Round 3**: Clarified question on whether patients limit sexual activity because of pain around vagina, and eliminated question on how often patients have pain inside or around vagina, as less interested in frequency versus whether or not they are actually limiting sex due to pain  **Round 4**: Patients tended to consistently respond to all versions but 1 patient with issues with recall period only consistently answered version C in accordance with her sexual activity status. |
| **GI/Urinary Symptoms** | In the past 30 days, how often did you experience difficulty in starting your stream or emptying your bladder? | In the past 30 days, how often did you experience difficulty emptying your bladder? | [no change from Round 2] | [no change from Round 3] | **Round 1:** Starting/emptying thought of as 2 separate concepts: Starting a stream versus having an unfinished feeling; unclear what to answer who has trouble emptying but not starting stream |
| **GI/Urinary Symptoms** | In the past 30 days, how often did you experience urine leakage related to coughing, sneezing or laughing? | [no change from Round 1] | [no change from Round 2] | [no change from Round 3] | N/A |
| **GI/Urinary Symptoms** | In the past 30 days, how often did you experience urine leakage associated with a feeling of urgency; that is, a strong sensation of needing to go to the bathroom? | [no change from Round 1] | In the past 30 days, how often did you experience urine leakage associated with a strong sensation of needing to go to the bathroom? | [no change from Round 3] | **Round 1:** No changes.  **Round 2:** Simplified question as ‘associated with feeling of urgency’ and ‘a strong sensation of needing to go to the bathroom’ were deemed redundant. ‘Urine leakage’ was underlined to emphasize the presence of leakage of urine related to these symptoms. |
| **GI/Urinary Symptoms** | In the past 30 days, how often after a bowel movement did you feel unfinished - that is, that you had not passed all your stool? | In the past 30 days, how often after a bowel movement did you feel unfinished - that is, that you had not passed all your stool?    [added as an option]:  In the past 30 days, how often have you had to return to the bathroom at least once in order to complete a bowel movement? | In the past 30 days, how often after a bowel movement did you feel unfinished - that is, that you had not passed all your stool? | [no change from Round 3] | **Round 1**: No significant issues with understanding the question; some interpreted as constipation  **Round 2:** Tested additional question on ‘having to return to the bathroom at least once’ to parse out defecatory dysfunction versus constipation  **Round 3**: Given participants answered similarly to both questions (though first question more likely to pick up on transit-related constipation in addition to defecatory dysfunction), agreed that new question added little |
| **Physical Function** | In the past 30 days, how much did a vaginal bulge limit you in doing your usual work including household chores? | In the past 30 days, how much did a vaginal bulge limit you in doing your usual work including household chores? | In the past 30 days, how much did you need to modify your usual work (including household chores) because of a vaginal bulge? | [no change from Round 3] | **Round 1:** Some had issues with word ‘limit’ as would otherwise push themselves through thus not technically limited.  **Round 2:** Some discussion about whether another word (change, adapt, affect) might be better as chores will take place either way; but overall decided to keep wording as participants generally understood the question and to harmonize this with the subsequent question on physical activities.  **Round 3:** Changed to ‘modify’ as this reflects that work such as chores ultimately get done (thus not limited) but modifications may have to be made to accomplish this |
| **Physical Function** | In the past 30 days, how much did a vaginal bulge limit you in doing activities, such as walking, running, participating in sports? | [no change from Round 1] | [no change from Round 2] | [no change from Round 3] | **Round 1:** No significant changes needed; overall well understood |
| **Pain** | In the past 30 days, how much did pain *in the shaded region* of your body limit your day to day activities?  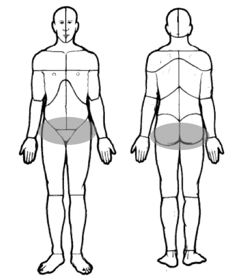 | In the past 30 days, how much did pain *in the shaded region* of your body limit your day to day activities?  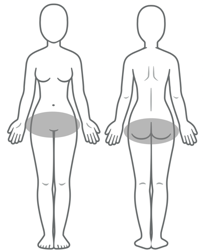 | [no change from Round 2] | [no change from Round 3] | **Round 2:** Some had issue with ‘limit’ with potential preference for ‘interfere’ or ‘modify’ as limit meant ‘restrict’ whereas what is experienced is discomfort during these activities.  Changed body map to reflect feminine figure |

Abbreviation: POP, pelvic organ prolapse.
